# Brazilian Green Propolis as a Therapeutic Agent for the Post-surgical Treatment of Caseous Lymphadenitis in Sheep

**DOI:** 10.3389/fvets.2019.00399

**Published:** 2019-11-26

**Authors:** Mauricio Alcântara Kalil, Laerte Marlon Santos, Thiago Doria Barral, Daniela Méria Rodrigues, Neila Paula Pereira, Maria da Conceição Aquino Sá, Marcelo Andres Umsza-Guez, Bruna Aparecida Souza Machado, Roberto Meyer, Ricardo Wagner Portela

**Affiliations:** ^1^Instituto de Ciências da Saúde, Universidade Federal da Bahia, Salvador, Brazil; ^2^Faculdade de Farmácia, Universidade Federal da Bahia, Salvador, Brazil; ^3^Centro Universitário SENAI-CIMATEC, Instituto de Tecnologias da Saúde, Salvador, Brazil

**Keywords:** anti-microbials, *Corynebacterium pseudotuberculosis*, natural products, sheep, wound healing

## Abstract

As antibiotics are ineffective when used to treat caseous lymphadenitis, the surgical excision of lesions is often required. Iodine solution (10%) is currently the choice for the post-surgical treatment; however, it may cause histotoxicity. Propolis are resinous substances composed by a mixture of different plants parts and molecules secreted by bees. As green propolis has already proven to possess anti-bacterial and wound healing properties, this study aimed to evaluate the use of a green propolis-based ointment as a therapeutic agent for the post-surgical treatment of caseous lymphadenitis. The caseous lesions of 28 sheep were surgically excised before dividing animals into two groups: (1) iodine-treated animals and (2) sheep treated with an ointment made with a previously characterized green propolis extract. Clinical data of animals, size of the scar area, the presence of moisture and secretion in the surgical wound, the humoral immune response against the bacterium and the susceptibility of *C. pseudotuberculosis* clinical isolates to the green propolis extract were analyzed. The green propolis-treated group presented complete healing of the surgical wound 1 week before the iodine-treated group. Additionally, animals treated with the green propolis ointment had fewer cases of wound secretion, but it was not statistically different from the iodine-treated group. No clinical signs indicating green propolis toxicity or other side effects were found, associated with a faster and more organized hair recovery by propolis use. The green propolis extract was able to inhibit the growth of 23 from the 27 *C. pseudotuberculosis* clinical isolates, with minimum inhibitory and minimum bactericide concentrations ranging from 01 to 08 mg/mL, and did not interfere with the humoral immune response against the bacterium. In addition, green propolis was able to inhibit biofilm formation by four of the *C. pseudotuberculosis* clinical isolates. We concluded that green propolis is a promising therapeutic agent to be used in the post-surgical treatment of caseous lymphadenitis in small ruminants due to its effects on surgical wound healing, hair recovery, inhibition of wound contamination and bacterial growth.

## Introduction

Caseous lymphadenitis (CL) is a chronic infectious disease that affects goats and sheep nearly on every continent. Its etiological agent is the bacterium *Corynebacterium pseudotuberculosis*, which can infect various species, including humans. However, little is known about the disease progression in humans as only a small number of cases have been previously reported (1). The transmission of this etiologic agent occurs through caseous secretions when in contact with mucous membranes or skin lesions that may arise due to shearing (2, 3). Although CL has low mortality, it causes economic losses, such as the devaluation of skin and carcass.

Conventionally, CL is treated via surgical drainage of the granulomatous lesions and subsequent chemical cauterization with iodine tincture (2, 4). Iodine treatment is known to be cytotoxic and is not 100% effective, and can disrupt the healing process (5). Administration of antibiotics does not produce a significant effect, and these agents are rather costly (6, 7).

Propolis is a resinous substance synthesized by bees using products present on floral buds, gems, and vegetable cuts. Besides being used as a material for repair, isolation, fixation, and microbiological protection of hives, its use as a therapeutic for several diseases has already been reported (8). Green propolis contains a significant amount of Artepillin C, a cinnamic acid derivative (9, 10). Artepillin C acts as an anti-inflammatory and anti-bacterial component when combined with other compounds present in green propolis (11). When it was first isolated and identified, it was found to have a significant anti-microbial potential (12). The anti-bacterial action of propolis is mainly demonstrated on Gram-positive bacteria (13) and has been tested as a major component of many pharmacological compounds used for various purposes, such as wound healing, anti-inflammatory action, and anti-bacterial activities (14).

The market for organic products was estimated to be ~90 billion dollars in 2016 (15). In organic animal production, Brazilian legislation allows the use of some natural substances in the therapeutic process. Green propolis, besides having a less pronounced anti-bacterial activity than red propolis, proved to have a better wound healing effect (16), and a lower cytotoxic potential (17, 18). In this context, the present study aimed to investigate the applicability of green propolis as a therapeutic agent in the post-operative treatment of CL.

## Materials and Methods

### Animals and Ethical Aspects

The experiment was carried out on a farm in the Monte Santo municipality, Bahia State, Brazil (10°24′12.7″S, 39°17′36.3″W). The owners of this extensive goat and sheep farm volunteered to look after the animals during the trial period.

Twenty-eight mixed-breed sheep of both sexes with clinical signs of superficial CL were used. These animals were transported to the farm 15 days before the beginning of the experiment and dewormed with albendazole 3.5 mg/kg. During these 15 days and throughout the experimental period, animals were fed with buffel grass pasture, daily supplementation of corn silage, soybean and crushed corn, and mineral salt and water *ad libitum*. The animals were randomly divided into two groups and were identified by the color of their earrings: a control group (Group 1) with 15 animals treated with 10% iodine tincture after surgery, and the test group (Group 2) with 13 animals treated with the green propolis-based ointment.

This study was approved by the Ethics Committee for the Use of Animals in Experimentation at the Veterinary School of the Federal University of Bahia (UFBA), under the protocol number 89/2017.

### Ethanolic Extract of Green Propolis and Ointment Production

The propolis extract was prepared as previously described (10). Fifteen milliliters of ethanol (80%) was added to 2 g of green propolis. Extraction was carried out at 70°C for 30 min under constant shaking at 710 rpm. Centrifugation at 8,000 × g for 10 min at 5°C (Centrifuge SIGMA 2-16 KL) was then performed and 10 mL of 80% ethanol was added prior to a second centrifugation step. The supernatant was homogenized and held at 50°C until completely dry. The extracts were then stored in tubes covered with aluminum foil under inert atmospheric conditions (N_2_) to avoid degradation. The characteristics and composition of the green propolis extract herein used are described in the Supplementary Material 1.

For the post-surgical treatment of CL, an ointment was prepared using green propolis, natural waxes, and oils. In the first step of ointment production, the oils and waxes (solid and liquid vaseline, lanoline, cera alba, cetostearyl alcohol, cholesterol) were blended and heated at 60°C until homogeneous and all ingredients had fused. The emulsion was then cooled to room temperature (25°C) with constant mixing to maintain the homogeneity. In the second step of ointment production, the green propolis extract (20% part-to-part) was added gradually to the mixture at room temperature under constant stirring. The final ointment appeared as a green oil/water cream that was easily applied, leaving an oily film on the skin surface.

### Surgical and Post-surgical Treatment

On the first day of the experiment, trichotomy was performed in the region of the affected lymph node. A 2.0-cm vertical incision was then made for the removal of the caseous content; this content was collected in sterile collectors. Samples were used to confirm the presence of *C. pseudotuberculosis* through microbiological examinations. The surgical procedure herein described is represented at the Supplementary Material 2.

In group one animals, the lymph node was drained and then internally cleaned with a sterile gauze soaked in 10% iodine dye. In group two, internal cleaning was performed with physiological solution prior to filling the cavity with the green propolis-based ointment (Supplementary Material 3). In both groups, after treatment, a repellent larvicidal spray was applied to the surgical incision. Post-operative treatment was performed only once on the first day in both groups, and the animals were observed for 2 months.

Before the surgical procedure and on a weekly basis during 2 months, a jugular venipuncture was created for blood collection. Ten milliliters of blood was collected in Vacutainer®-type tube without anti-coagulant for serum samples obtaining. Additional 10 mL of blood were collected in tubes with heparin anti-coagulant for clinical biochemistry assays.

### Serology

Serum samples obtained from sheep were immunologically assessed by an indirect ELISA to detect anti-*C pseudotuberculosis* specific IgG antibodies, as previously described (19).

### Clinical Parameters

Before the surgical procedure and on a weekly basis over the 2 months, clinical evaluations such as body condition score, respiratory (RR) and cardiac rates (RH), rectal temperature (RT) (in degrees Celsius), degree of anemia by via conjunctiva staining assessment, degree of hydration through the skin turgor test, and palpation of other superficial lymph nodes were performed. The lesion scars were measured weekly using a pachymeter. The presence of secretion and humidity in the lesions was also assessed.

### Clinical Biochemistry Evaluation

Plasma components of animals were evaluated by clinical biochemical analyses using commercial kits (Labtest). These analyses included the evaluation of uric acid, urea, creatinine, total proteins, ALT, and AST.

### Susceptibility of *C. pseudotuberculosis* Clinical Isolates to Green Propolis Extract

Caseous samples collected during the surgical procedure were subjected to a bacterial culture in blood agar medium to isolate and identify *C. pseudotuberculosis*. After confirming the species of the isolates through biochemical proofs, their susceptibility profile was determined by the microdilution assay to determine the minimum inhibitory concentration (MIC) and minimum bactericide concentration (MBC). For isolation, the granuloma material obtained from diseased animals was seeded on blood agar supplemented with 5% sheep blood. The agar plates were incubated (2% CO_2_) for 48 h at 37°C. The isolated colonies were macroscopically characterized and stained by the Gram method. Final identification of the isolates was performed by biochemical tests for catalase production, carbohydrate fermentation, urease production, and motility. To evaluate MIC and MBC, the green propolis ethanolic extract was diluted in 1% DMSO. Concentrations in the range of 0.016 to 8 mg/mL were tested, and the diluent control was also assessed. The MIC methodology was performed according to a previous report (20) with some modifications. Clinical isolates were grown in BHI broth 0.1% Tween at 37°C for 24 h. Each clinical isolate was then diluted in BHI broth to achieve an optical density (OD) of 0.08–0.10 at a wavelength of 600 nm (optically comparable to the McFarland standard). Each bacterial suspension had ~3 × 10^6^ colony forming unit (CFU)/mL of *C. pseudotuberculosis*. The suspensions were immediately diluted in BHI broth to obtain a concentration of 1 × 10^6^ CFU/mL. Each well was inoculated with 100 μL of the inoculum and 100 μL of a solution containing the green propolis extract. Plates were incubated at 37°C for 48 h and the bacterial growth was assessed with a plate reader set at 600 nM. MIC_100_ was defined as the lowest anti-microbial concentration capable of totally preventing microbial growth in 48 h.

A 20-μL aliquot from each well was inoculated on the surface of BHI agar and incubated at 37°C (2% CO_2_) for 48 h. MBC_100_ was defined as the lowest anti-microbial concentration that caused total bacterial death. These assays were performed in duplicate.

### Inhibition of Biofilm Formation by Green Propolis Extract

The quantitative analysis of biofilm production followed the methodology described by Merino et al. (21) with minor modifications. The *C. pseudotuberculosis* clinical isolates were inoculated in 3 mL of Tryptone Soya Broth (TSB) and incubated at 37°C until obtaining an optical density (OD) of 0.2 at 595 nm wavelength. Then, 200 μL of this bacterial suspension was transferred to sterile microplates and incubated at 37°C for 48 h. After incubation, the contents of each well were aspirated and the wells were washed twice with 200 μL of 0.01 M PBS pH 7.2. The bacteria that remained adhered were fixed with 200 μL of methanol and left in the incubator until dry. The wells were then stained for 5 min with 200 μL of a 2% crystal violet solution and then washed six times with distilled water. The dye impregnated into the adherent bacterial cells was then eluted with 160 μL of a 33% acetic acid solution. As negative control for this test, we used wells with sterile soybean broth (TSB) and no inoculum, and as positive control, the biofilm-producing *C. pseudotuberculosis* CAPJ4 strain (GenBank: CP026499.1) (22). The OD of each well was measured at a wavelength of 595 nm. To characterize the intensity of biofilm formation, the following equations were used, where ODI states for the optical density of the isolate and ODNC stands for optical density of the negative control: ODI ≤ ODNC = no development of biofilm; ODI / ODNC ≤ 2 = weak formation of biofilm; ODI / ODNC ≤ 4 = moderate capacity to produce biofilm; ODI / ODNC > 4 = strong capacity to produce biofilm.

To evaluate the ability of green propolis to interfere on biofilm formation by *C. pseudotuberculosis*, the isolates that were able to form biofilm and the CAPJ4 strain were selected. One hundred microliters of the bacterial suspensions with an OD of 0.2 and 100 μL of green propolis extract in different concentrations were inoculated in the wells of a sterile microplate and incubated at 37°C for 48 h. Then, the formed biofilm was detected as described above, and the percentage of biofilm production inhibition was calculated considering the control bacterial suspensions that were not incubated with green propolis.

### Mathematical and Statistical Analysis

All data obtained were analyzed using GraphPad Prism v.6 software. Student's *t*-test was used to determine significant differences between the means of the surgical wound area and OD means from the serological evaluation, RH, RR, body temperature, and clinical biochemistry values. Odds ratio test was used to verify the existence (or lack thereof) of significant statistical differences between the groups for moisture or purulent secretion presence in the surgical wounds.

## Results

### Wound Healing

**Figure 1:**
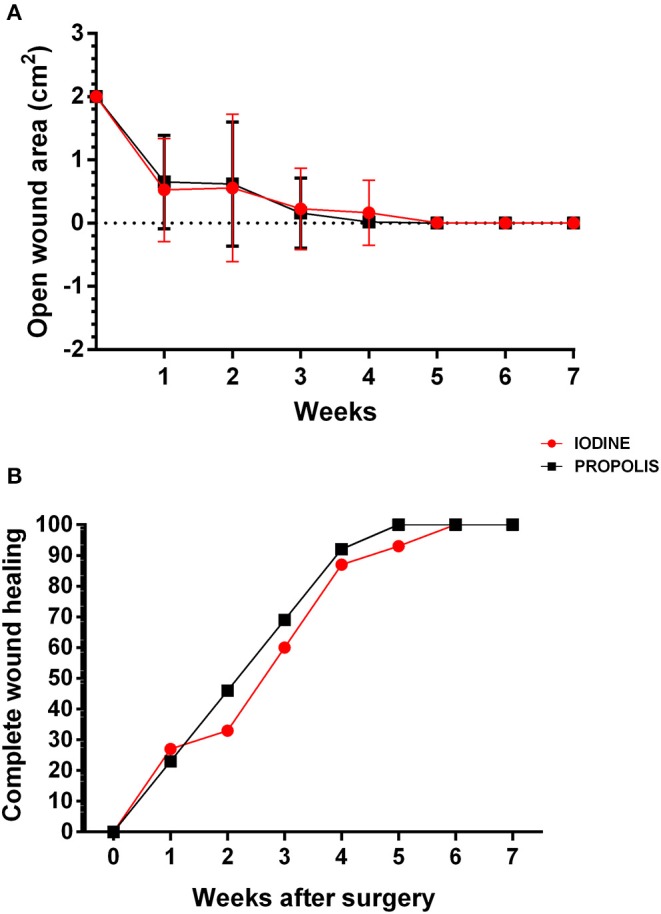
Wound healing process in animals treated with iodine or green propolis-based ointment. **(A)** Open wound area 2 weeks after surgery. Averages of the open wound area in each experimental group over the weeks are presented, and bars represent their standard deviations. The results of the two groups at each time were compared by Student's *t*-test with *p* < 0.05; no significant differences were found. **(B)** Percentage of animals from each experimental group with complete wound healing. The points on the chart show the percentage of animals in each group achieving complete healing over the weeks.

The values related to the cicatricial process are shown in Figure 1A. Based on the mean values for the surgical wound areas in both experimental groups, significant differences were not found throughout the experiment. Animals treated with green propolis had a fully healed surgical incision 1 week before animals treated with iodine (Figure 1B). It should also be emphasized that the animals treated with propolis had a faster and organized hair recovery process (Supplementary Material 4), which was not observed in animals treated with iodine.

**Figure 2:**
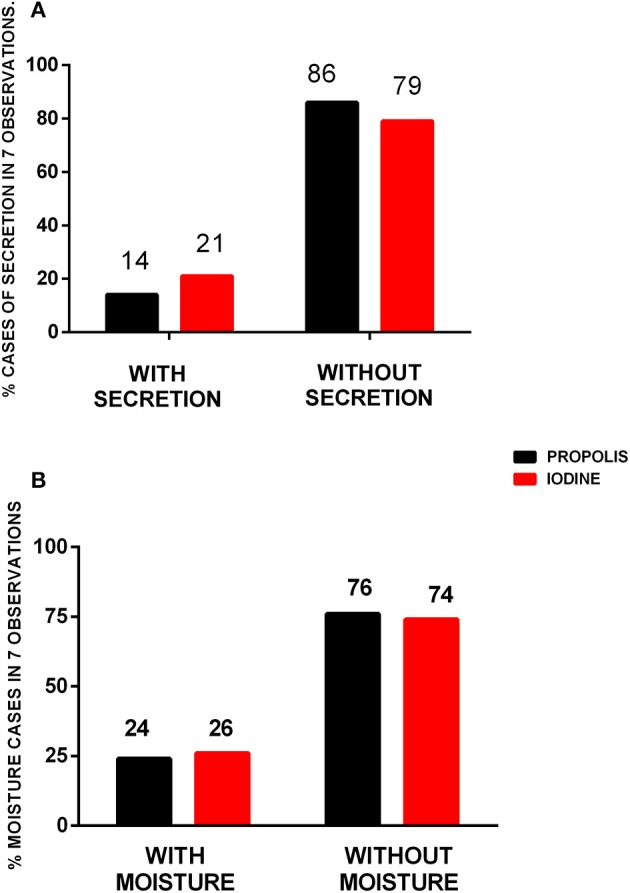
Cases of purulent secretion and moisture in surgical lesions after treatment with propolis or iodine. Percentage of purulent secretion **(A)** and moisture **(B)** cases in the surgical wounds in seven observations throughout the 2-month observation period. Data were compared by the odds ratio test, with *p* < 0.05; no significant difference was found between them.

Of the animals treated with 10% iodine tincture, 21% had purulent secretion. In the green propolis ointment group, however, this was only present in 14% of the population (Figure 2A). The same result was found due to moisture in the lesions; 24% in the ointment-treated group and 26% in the iodine tincture-treated group (Figure 2B). Based on the odds ratio, however, no statistical difference was found between the groups for both parameters.

### Clinical Evaluation

**Figure 3:**
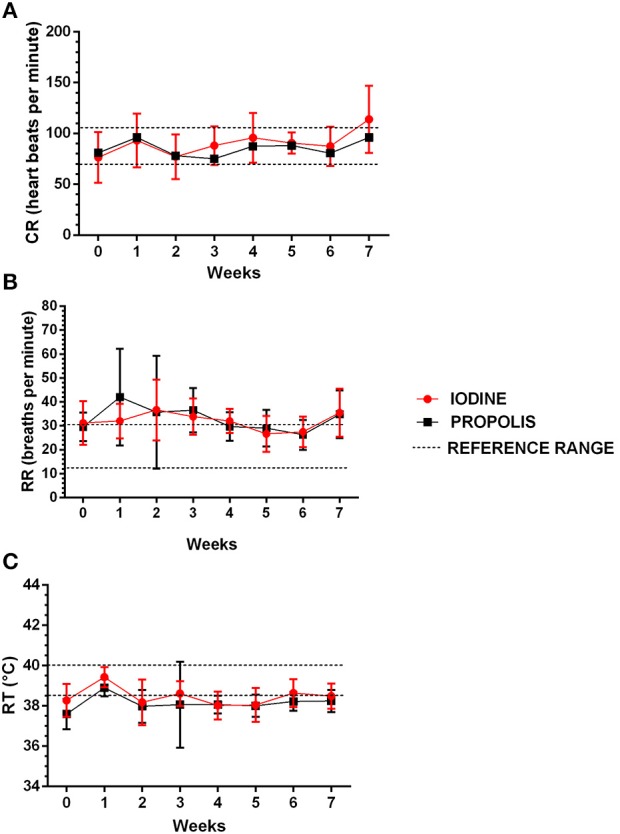
Clinical parameters of the animals treated with iodine and green propolis ointment after excision of the caseous lymphadenitis lesion. The parameters evaluated were **(A)** cardiac rate, **(B)** respiratory rate, and **(C)** rectal temperature. The data are presented as mean, and bars indicate their respective standard deviations. The dashed lines correspond to the reference range (2, 24). There were no statistically significant differences between the two experimental groups at any time in any of the parameters, by the Student *t*-test (*p* < 0.05).

**Figure 4:**
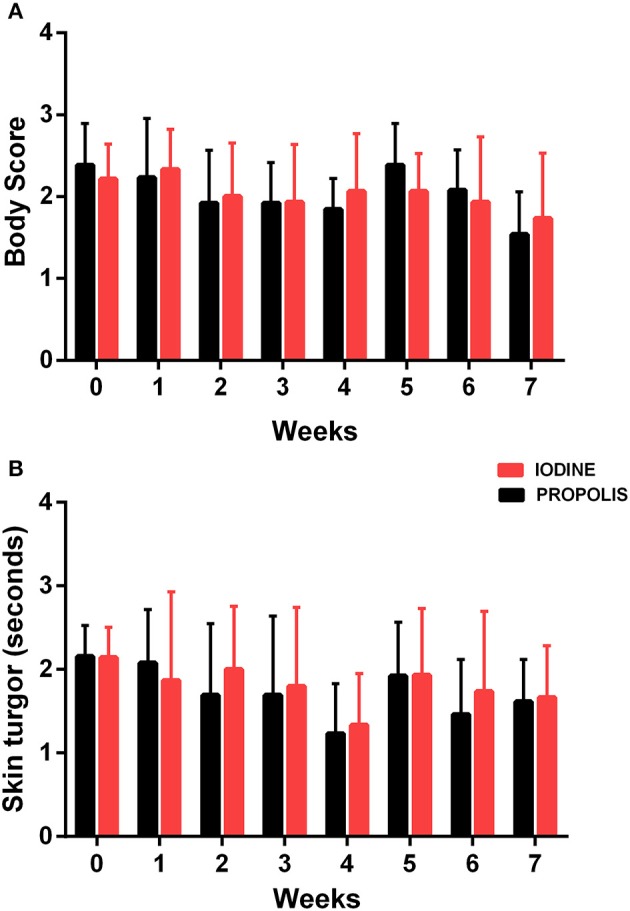
Determination of **(A)** body condition score and **(B)** time of return of skin turgor in experimental animals. Results are presented as mean at each observation time for each group, and bars indicate their respective standard deviations. There were no statistical differences by the Student *t*-test (*p* < 0.05) for any of the observation times.

Animals were evaluated between March and May, when the temperature at the farm ranged from 31 to 34°C (23). Throughout the experiment, cardiac rate (RH), respiratory rates (RR), and rectal temperature (RT) for the two groups slightly varied, with no statistical difference between the experimental groups (Figures 3A–C). Mean body condition scores (Figure 4A) and average hydration levels (Figure 4B) did not statistically differ between the two groups.

**Figure 5:**
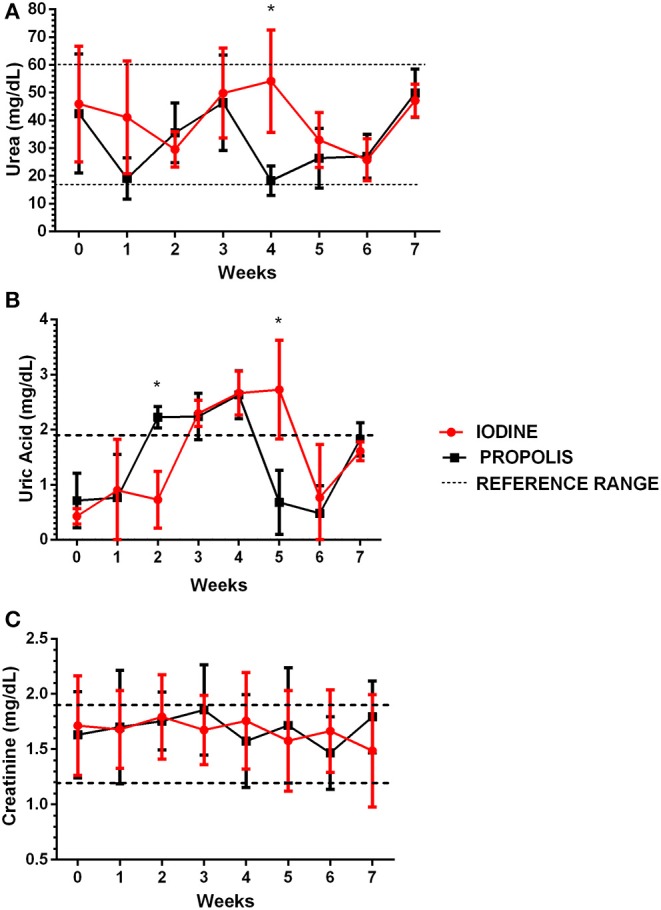
Determination of urea **(A)**, uric acid **(B)**, and creatinine **(C)** in serum samples from animals in the two experimental groups throughout the experiment. The results are presented as mean of each observation time for each group, and the bars indicate their respective standard deviations. The dotted lines represent the reference ranges for urea (27), uric acid (28), and serum creatinine (27). Data were compared by the Student *t*-test, with *p* < 0.05; no significant difference was found between them.

Plasmatic urea remained statistically similar between the two experimental groups, except for the 4-week post-surgery results (Figure 5A) where values for the iodine group were statistically different from those of the propolis group, but within normal reference values (25). Values for serum uric acid levels (Figure 5B) were slightly above the upper reference level (26), with differences between groups at two points, 2 and 6 weeks after surgery. Nonetheless, values were still slightly above the reference maximum value. Serum creatinine levels were within normal limits (25) and without statistical significant differences when the two groups were compared throughout the experiment (Figure 5C).

**Figure 6:**
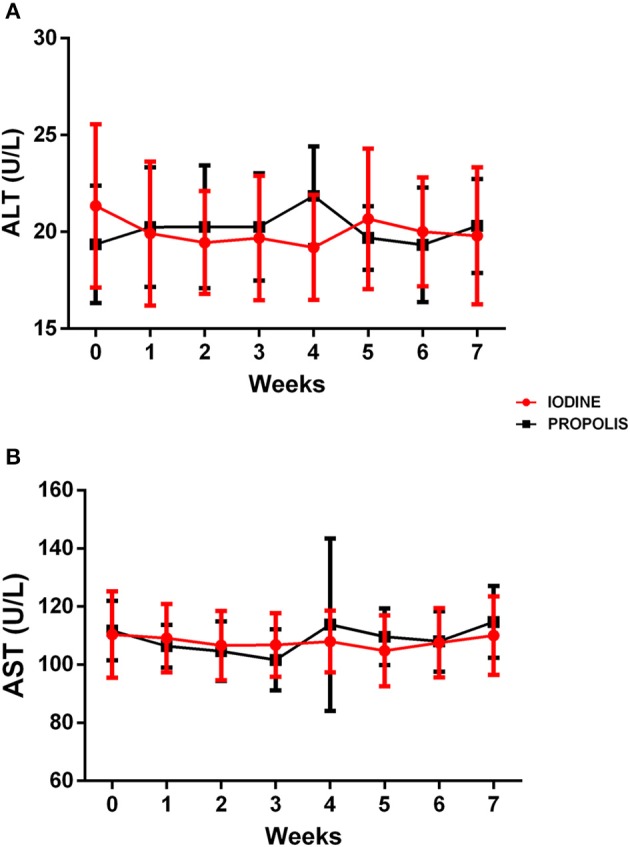
Determination of ALT **(A)** and AST **(B)** levels in animals of the two experimental groups throughout the observation period. Results are presented as mean of each observation time for each group, and bars indicate their respective standard deviations. There were no significant statistical differences between the groups when the Student *t*-test (*p* < 0.05) was used to compare the mean of each group at a given time.

The results of serum total protein levels found in the present study are within the normal parameters (25) and no statistical differences were found between the experimental groups (data not shown). The levels of hepatic enzymes, alanine aminotransferase (ALT) and aspartate aminotransferase (AST) (Figures 6A,B) in the two experimental groups remained very close throughout the experiment and without significant statistical differences. To add, they remained within the normal patterns indicated in the literature (25).

### Serology

**Figure 7:**
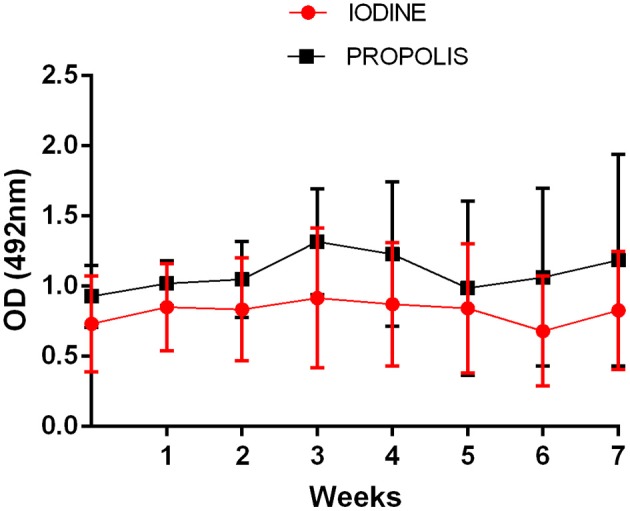
Production of anti-*C pseudotuberculosis* specific antibodies in animals treated with iodine or green-propolis-based ointment. Data are shown as mean of the optical densities (OD) values, and bars represent their respective standard deviations. No statistical differences by the Student *t*-test (*p* < 0.05) were found between the two groups at the same collection time.

All animals had a positive serological test for *Corynebacterium pseudotuberculosis*-specific antibodies detection by indirect ELISA. Throughout the experimental period, the levels of specific antibodies were not significantly different between the two groups (Figure 7).

### *C. pseudotuberculosis* Susceptibility to the Green Propolis Extract

Twenty-seven clinical isolates of *C. pseudotuberculosis* could be retrieved from the animals included in this study, and the sensitivity test could not be performed with the caseous material from one of the animals. Although identified as *C. pseudotuberculosis*, this isolate was later lost due to difficulties in its activation and growth. The concentrations of 1.0 and 2.0 mg/mL of the green propolis ethanolic extract most frequently inhibited 100% of the bacterial growth, inhibiting 52% of isolates. The concentration of 8.0 mg/mL inhibited 30% of the isolates, while 15% showed no sensitivity to the concentrations of green propolis herein tested (Table 1). The minimum bactericidal concentration (MBC) data of the isolates are presented in Table 1. Forty-eight percent of the isolates presented MBC at concentrations of 1.0 to 2.0 mg/mL, while 33% had MBC at 8.0 mg/mL (Table 1). The green propolis extract tested concentrations could not display any bactericidal action against six of the isolates.

**Table 1:** Sensitivity of *C. pseudotuberculosis* clinical isolates obtained from sheep with caseous lymphadenitis to green propolis extract.

**Isolate** **ID**	**MIC_**100**_** **(mg/mL)**	**MBC_**100**_** **(mg/mL)**
03	>8	>8
04	>8	>8
05	8	8
06	2	2
10	4	4
11	1	1
12	1	1
13	1	1
14	8	>8
17	8	>8
18	1	2
19	1	2
20	1	1
52	2	2
54	2	2
55	1	1
56	2	2
58	1	1
60	1	1
61	8	8
62	1	1
64	8	8
80	4	4
81	8	8
89	>8	> 8
94	>8	> 8
98	2	2

*We obtained 27 isolates from the 28 animals included in this experiment. The minimum inhibitory concentration of green propolis that could inhibit the growth of 100% of bacteria (MIC_100_) and the minimum bactericidal concentration that could kill 100% of the bacteria (MBC_100_) were obtained using the microdilution method*.

### Inhibition of Biofilm Formation

**Figure 8:**
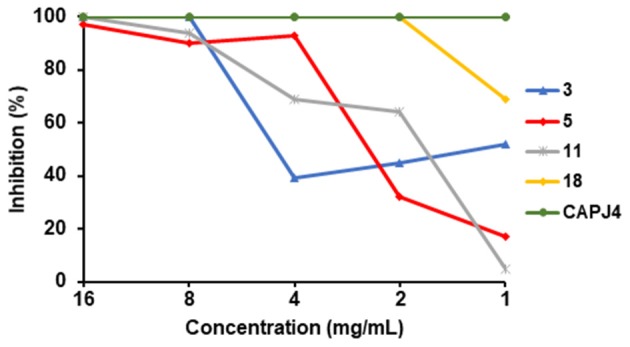
Inhibition of *C. pseudotuberculosis* biofilm formation by green propolis extract. Four *C. pseudotuberculosis* clinical isolates and the CAPJ4 biofilm producing strain were incubated with different green propolis extracts concentrations for 48 h, and biofilm was then quantified. Each line represents the results of an individual *C. pseudotuberculosis* biofilm-producing strain. Results are expressed as means of three independent experiments.

It was observed that 23 from the 27 clinical isolates were negative for biofilm formation. Of the four biofilm producing isolates, one showed moderate production and three isolates showed poor biofilm formation. The control CAPJ4 strain showed a strong capacity to form biofilm. Green propolis extract at the concentrations of 16 and 8 mg/mL was able to fully prevent biofilm formation by three isolates, having greater efficacy in one of them, where the concentration of 2 mg/mL was able to totally inhibit the formation of biofilm. One of the isolates did not show 100% inhibition of biofilm formation, presenting a 97% inhibition when incubated with the highest concentration of green propolis herein investigated. Interestingly, the positive control, the *C. pseudotuberculosis* CAPJ4 strain, which had a strong biofilm formation, presented 100% inhibition of biofilm formation at all tested propolis concentrations (Figure 8).

## Discussion

The treatment of caseous lymphadenitis in small ruminants has been a matter of discussion among veterinarians, since commercial antibiotics are not effective against the disease, and the currently used iodine tincture can present histotoxicity and a delay in the animals' hair recovery. In this way, we proposed in this study a new topic treatment represented by a Brazilian green propolis-based ointment, with promising results.

More than 14 types of propolis were already described in Brazil and classified according to their physical-chemical properties and geographic localization (4). From these, red propolis has already proved to have a pronounced anti-bacterial activity against Gram positive and Gram negative bacteria (10), however it presented a higher cytotoxic potential than green propolis (17, 18). Indeed, green propolis has already proven to have a significant wound healing activity, and also has anti-bacterial activity. In this way, green propolis was chosen to be used in this work for the ointment production.

When the cicatricial process was evaluated after surgical incision in Wistar rats treated with honey and brown propolis, no statistical difference was found in the surgical wound areas in the two groups when compared to the control group. However, a better induction of healing was observed in the propolis-treated group based on the histological profile of the lesions (29). In another experiment performed with Wistar rats, no statistical difference was observed for wound healing using green tea cream, a 20% propolis ointment and saline solution as control (30). Likewise, in a healing test performed with the propolis ointment compared to the ointment base alone in rats, a higher healing rate was found in the group treated with propolis (31). In a similar experiment with dogs, the ointment containing brown propolis could heal early surgical wounds (24). As these studies demonstrated faster healing with propolis, their results resembled those of the present study, as we were able to see that sheep treated with the propolis-based ointment were able to heal the wound 1 week before the iodine-treated group. This faster healing process can also be explained by the rapid absorption of propolis, as has been observed in studies with subcutaneous implants containing green propolis (32, 33).

In this study, other parameters such as the presence of secretion and moisture in the lesions were used to evaluate healing, in a similar way to a study where the effect of propolis on healing was determined (30). The lower occurrence of purulent secretions in the surgical wounds of animals treated with the green propolis-based ointment than in those treated with 10% iodine tincture could indicate less infection of wounds by opportunistic agents or even an anti-bacterial effect of the treatment against *C. pseudotuberculosis* present in granulomatous lesions. The anti-bacterial power of propolis has already been described in studies where very low minimum inhibitory concentrations (MIC) were observed for Gram-positive bacteria (10, 34). An anti-bacterial effect for Gram-negative bacteria, such as *Pseudomonas aeruginosa, Pseudomonas* sp., and *Escherichia coli*, has also been visualized and this type of bacteria is an important source for contamination of surgical wounds (13, 27, 34).

It is important to notice that we were able to see a faster and more organized hair recovery in the propolis-treated animals, when compared to the iodine-treated animals. This situation may be related to the histotoxic profile of iodine tincture (3), and is important when considering animals that are bred for expositions and competitions.

An increase in temperature in sheep has been reported to influence clinical parameters such as cardiac rate (RH), respiratory rates (RR), and rectal temperature (RT) (28). However, the procedures described herein were performed in a covered environment, at the same time of day, and under constant temperature, thereby minimizing the environmental influence on the measured parameters; values remained within the normal parameters presented in the clinical literature (35). For RT, stress caused by restraint during collection may have led to some of the averages being above normal in the first weeks, as has been demonstrated in other studies with sheep (36, 37). For hydration levels, these results were within normal limits described for the Brazilian semi-arid region (38); however, body condition score was below values considered normal for the species in both groups (39); the latter results can be explained as we used mixed breed animals maintained under extensive management in this experiment.

The absence of significant differences in the clinical parameters of the animals treated with propolis or iodine during the entire experiment demonstrates that green propolis does not induce clinically observable adverse side effects. A similar situation was observed in an experiment using intramammary green propolis in dairy cows for the treatment of mastitis (40). Indeed, there are also reports that green propolis can protect against ultraviolet radiation by serving as a sun protection factor (41).

Regarding the levels of serum uric acid found, diets with a higher crude protein level can induce a small increase in these levels (42). Varying feed composition, mainly by considering different batches of silage supplied during the experiment, could result in a small increase in uric acid levels in the second, third, and fourth weeks. The results also show that treatment with green propolis does not result in any hepatotoxic effects that can be observed through varying levels of total proteins (43).

In a study using green propolis as an adjuvant for vaccines, the ethanolic extract did not efficiently stimulate humoral immunity, but rather cellular immunity (44). In contrast, it was already described that green propolis can inhibit the recruitment and activation of neutrophils and macrophages, and also the production of Tumor Necrosis Factor (45). Doi et al. (46) observed that suppression of inflammation and oxidative stress in a murine model of colon tumorigenesis. Considering this situation, we looked after the kinetics of the specific humoral response against the bacteria in the observation period, with the objective to analyze if the treatment with green propolis would be able to affect the production of *C. pseudotuberculosis-*specific immunoglobulins, since a lower innate response can interfere with lymphocyte activation and the anti-phospholipase D immunoglobulins are important to limit the spread of the bacteria (19). In the present experiment, no effect on the levels of the humoral response specific to *C. pseudotuberculosis* were found, despite these previous reports on the anti-inflammatory activity of this propolis.

In previous studies with Gram positive bacteria, the methicillin-resistant *S. aureus* strains ATCC25923, ATCC33591, ATCC6538, and *Enterococcus faecalis* were inhibited by green propolis at concentrations of 0.4–0.2, 1.6–0.05, 0.2, 0.4, and 0.4 mg/mL respectively (10, 17); these values are lower than those found in the present study. However, in the prior studies, MIC was simply reported as the concentration that inhibited any bacterium growth, whereas in this study, MIC was defined as the concentration that completely inhibited bacterium growth. Similarly, previous studies comparing MIC also showed concentrations of 1.6–0.8 mg/mL for Gram-positive bacteria (10, 17). This higher resistance of *C. pseudotuberculosis* might be due to the structure of its cell wall. Although this species is a Gram-positive bacterium, the presence of a peptidoglycan bound layer on the polymer of arabinogalactan with mycolic acids in the outer membrane may serve as a mechanism of resistance developed by the *Corynebacterium* genus (47). The alcoholic extract of Taiwanese green propolis, with high concentrations of the flavonoids propoline C, D, F, and G had MIC of 0.01 to 0.02 mg/mL against *S. aureus* and 0.64 mg/mL against *E. coli*. However, only a few studies have been performed on these flavonoids in Brazilian green propolis extracts, and only little can be speculated on whether the presence of these compounds could have influenced or caused a different result (48).

Propolis extracts from different geographical origins and different types have been shown to have significant anti-microbial and biofilm inhibiting activities (49). In the present study, Brazilian green propolis extract was effective in interfering with biofilm formation by bacterial isolates obtained from the animals herein studied. Cardoso et al. (50) verified a significant inhibitory action of a green propolis extract rich in hydroxycinnamic acids in biofilm formation by *Streptococcus mutans*. Brown propolis extracts rich in prenylated phenylpropanoic acids were able to inhibit biofilm formation by *S. aureus* (51), a situation also observed by El-Guendouz et al. (52). Importantly, the composition of propolis is directly linked to its anti-biofilm effect, as seen by Duarte et al. (53) which were not able to observe inhibition of biofilm formation by *S. aureus* when analyzing propolis extract fractions without flavonoids.

To summarize, we could confirm that, compared to treatment with 10% iodine tincture, the application of the green propolis-based ointment is a better post-surgical treatment of CL in sheep. This was demonstrated by the 1-week reduction in the healing process. For livestock animals, this indicates an appropriate time where contaminations by opportunistic bacteria would occur. Also, it was observed a faster and more organized hair recovery. The *in vitro* sensitivity of the isolates collected from the green propolis ethanolic extract was also examined. Applying the green propolis ointment did not induce any observable collateral effect when the laboratory biochemistry data and treated animals were evaluated.

## Data Availability Statement

All datasets generated for this study are included in the article/Supplementary Material.

## Ethics Statement

This study was approved by the Ethics Committee for the Use of Animals in Experimentation at the Veterinary School of the Federal University of Bahia (UFBA), under the protocol number 89/2017.

## Author Contributions

MK, LS, TB, DR, NP, MS, and MU-G performed the experiments. MK, LS, TB, and RP performed the data analysis. MK, MU-G, and RP designed the experiments and wrote the manuscript. BM, RM, and RP critically reviewed the manuscript. All authors contributed to manuscript revision, read, and approved the submitted version.

### Conflict of Interest

The authors declare that the research was conducted in the absence of any commercial or financial relationships that could be construed as a potential conflict of interest.
