# Supplementary material for: Brazilian Green Propolis as a Therapeutic Agent for the Post-surgical Treatment of Caseous Lymphadenitis in Sheep
Source: Front Vet Sci. 2019 Nov 26;6:399. doi: 10.3389/fvets.2019.00399 (PMC6887654; doi:10.3389/fvets.2019.00399)
Supplement: Supplementary file 1 [file Data_Sheet_1.PDF]

Supplementary material 1 – Composition of the green propolis extract and its characteristics, as described by [10].

Parameters	Content
Humidity (%)	8.84 ± 0.05
Total solids (%)	91.16 ± 0.05
Total ash (%)	3.30 ± 0.11
Protein (%)	10.58 ± 0.08
Lipids (%)	45.76 ± 1.77
Water activity	0.705 ± 0.01
Fiber (%)	16.3 ± 1.34
Sodium (mg/Kg)	2.40 ± 0.01
Potassium (mg/Kg)	399.1 ± 4.91
Lithium (mg/Kg)	1.80 ± 0.01
Calcium (mg/Kg)	9.00 ± 0.01
Phenolic Compounds (mg EAG/g)	181.71±0.01
Flavonoids (mg EQ/g)	46.80±0.01
p-coumaric acid (μg/mL)	24.65±0.24
Artepillin C (μg/mL)	569.85±0.11
