# Supplementary material for: Brazilian Green Propolis as a Therapeutic Agent for the Post-surgical Treatment of Caseous Lymphadenitis in Sheep
Source: Front Vet Sci. 2019 Nov 26;6:399. doi: 10.3389/fvets.2019.00399 (PMC6887654; doi:10.3389/fvets.2019.00399)

Supplementary material 2 - Surgical procedures used in this study. Trichotomy **(A)**, measurement of the incision **(B),** incision with a sterile blade **(C),** and removal and collection of the caseous content for microbiological evaluation **(D).**

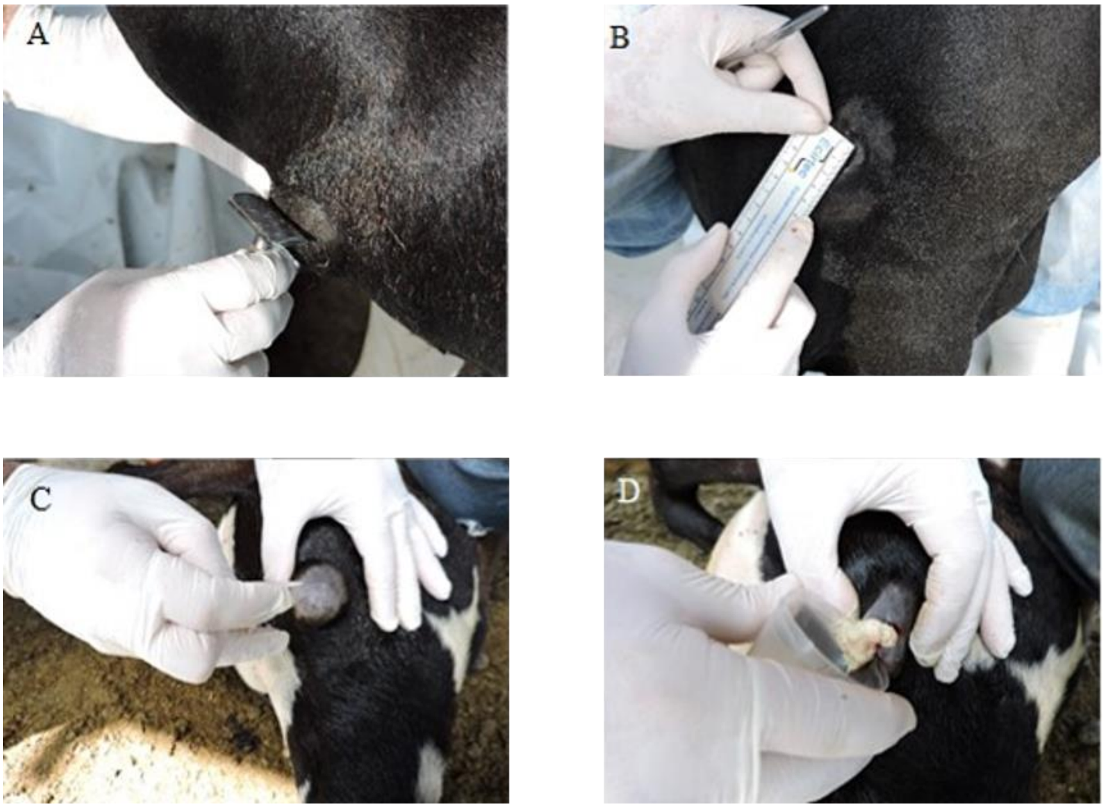

Supplement: Supplementary file 2 [file Data_Sheet_2.PDF]
