# Supplementary material for: Brazilian Green Propolis as a Therapeutic Agent for the Post-surgical Treatment of Caseous Lymphadenitis in Sheep
Source: Front Vet Sci. 2019 Nov 26;6:399. doi: 10.3389/fvets.2019.00399 (PMC6887654; doi:10.3389/fvets.2019.00399)

Supplementary material 3 – Application of green propolis ointment after surgical cavity cleaning (A), and treatment with 10% iodine and larvicide spray (B).

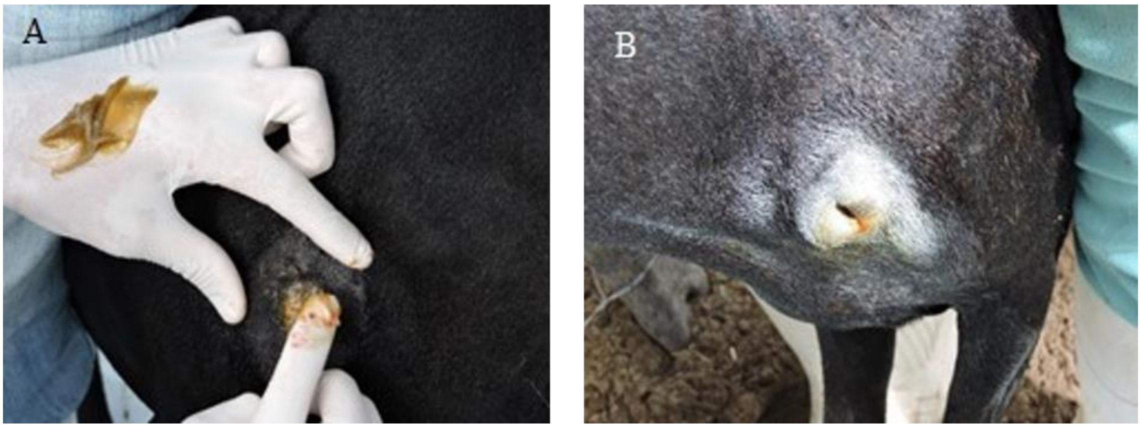

Supplement: Supplementary file 3 [file Data_Sheet_3.PDF]
