# Supplementary material for: Brazilian Green Propolis as a Therapeutic Agent for the Post-surgical Treatment of Caseous Lymphadenitis in Sheep
Source: Front Vet Sci. 2019 Nov 26;6:399. doi: 10.3389/fvets.2019.00399 (PMC6887654; doi:10.3389/fvets.2019.00399)

Supplementary material 4 - Hair growth around the surgical lesion. Normal growth of hair around the lesion treated with green propolis ointment **(A),** and absence of hair around the treated lesion with 10% iodine tincture **(B).** Images represent animals from different experimental groups observed at the same time after surgical excision of the granulomatous lesions.

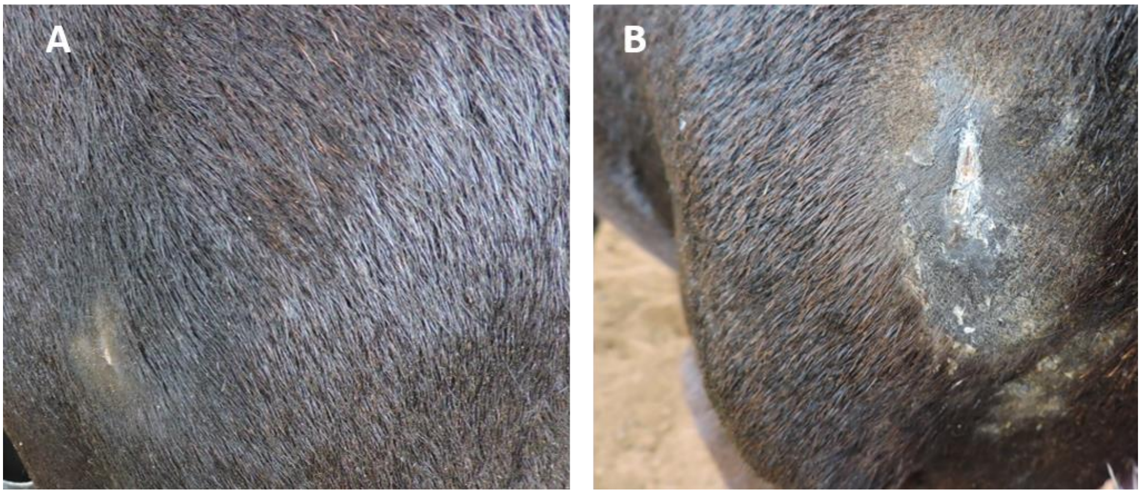

Supplement: Supplementary file 4 [file Data_Sheet_4.PDF]
